# The Behavior of Supersonic Jets Generated by Combination Gas in the Steelmaking Process

**DOI:** 10.3390/ma14175034

**Published:** 2021-09-03

**Authors:** Binglong Zhang, Fuhai Liu, Rong Zhu

**Affiliations:** 1 School of Metallurgical and Ecological Engineering, University of Science and Technology Beijing, Beijing 100083, China; aptx2008@126.com (B.Z.); zhurong1206@126.com (R.Z.); 2 National Center for Materials Service Safety, University of Science and Technology Beijing, Beijing 100083, China

**Keywords:** laval nozzle, supersonic jet, combination gas, flow field, numerical simulation

## Abstract

In the duplex steelmaking process, the oxygen flow rate is suppressed to reduce the increasing rate of the temperature in the molten bath, resulting in severe dynamic conditions. To improve the mixing effect of the molten bath, a Laval nozzle structure designed for combination gas has been proposed. In this research, five types of Laval nozzle structure have been built based on the combination gas content, and both numerical simulations and experiments are performed to analyze the flow field of the supersonic jet. The axial velocity and oxygen concentration were measured in the experiment, which agreed well with the numerically simulated data. The results show that both initial axial velocity and potential core length increase with the flow rate of combination gas. Further, applying a higher N_2_ flow rate could improve the oxygen utilization rate at different ambient temperatures, but this issue increases the oxygen utilization rate; however, the latter can be reduced at higher ambient temperatures.

## 1. Introduction

The duplex steelmaking process has been widely utilized to enrich valuable elements (vanadium and titanium), or to remove harmful elements (phosphorous) [1,2]. According to the operational characteristics, there are two common operational methods for the duplex steelmaking process. In traditional methods, such as the Linz–Donawitz (LD)-new refining process (NRP) and the simple bearing process (SRP) method, the hot metal is first discharged into a dephosphorization converter for producing the low phosphorus semi-steel. Subsequently, the semi-steel is discharged into a decarburization converter to produce the molten steel, which meets the requirements of content and temperature [3,4]. For the multi-refining converter (MURC) method, the liquid slag is kept inside the converter after the decarburization process and is then reused in the dephosphorization process in the same converter, which decreases the slag quantity and smelting cost [5]. To achieve a better thermodynamic condition and an appropriate molten time, oxygen flow with a lower rate should be utilized to lessen the reaction rate between the oxygen and carbon elements [6,7]; nevertheless, the mixing and impaction ability of supersonic oxygen multi-jets can also be reduced, which suppresses the dynamic conditions of the molten bath [8,9].

For refining the dynamic condition of the molten bath, the influences of the Laval nozzle structure, oxygen initial temperature, and injection angle on the flow field of the supersonic jet have been broadly examined by both numerical simulation and experiment, at the same conditions of the oxygen flow rate. Wang and Liu et al. [10,11] reported that the oxygen preheating technology could significantly improve the initial velocity of the supersonic jet, resulting in a more considerable impaction ability. Sambasivam and Feng et al. [12,13] proposed a new arrangement for the oxygen nozzles, proved that an added center nozzle could increase the impaction cavity of the molten bath, and demonstrated the flow field of the oxygen multi-jets. Wang et al. [14] investigated the behavior of supersonic multi-jets generated by an oxygen lance designed with three large-flow-rate Laval nozzles and three small-flow-rate Laval nozzles. They reported the smaller Laval nozzle had a more significant impact on the cavity area than the larger Laval nozzle. Odenthal et al. [15,16] designed a series of Laval nozzle structures by various methods and analyzed the effect of boundary layer parameters of the Laval nozzle wall on the shock wave shape and potential velocity length, which further proved that the characteristic-line design method could restrict the formation of the expansion wave at the Laval nozzle exit. Although the mentioned research works optimized the flow field during the oxygen jet development, the intensity of the top-blowing gas supply was still not enough in the vanadium extraction or the dephosphorization process. In recent years, Wei and Wu et al. [17,18] evaluated the CO_2_ and O_2_ mixed injection method in electric arc furnace (EAF) steelmaking and described the flow field distribution of the supersonic jet using different CO_2_ mixing rates. The oxidation reaction between the oxygen and the elements in the molten bath can be suppressed by adding an inert gas into the pure oxygen jet, particularly at a larger top-blowing flow rate.

However, the pure CO_2_ gas increases the operational cost of the steelmaking process. At the same time, for producing the O_2_ gas, N_2_ gas will also be generated during the gas separation. Meanwhile, with the extra N_2_ gas flow at the top-blowing process, the temperature at the impaction cavity will be suppressed, which improves the vanadium extraction rate and dephosphorization rate, according to thermodynamics theory. Moreover, the mixed injection method using the O_2_ and N_2_ also enhances the dynamic condition in the molten bath, which further increases the vanadium extraction rate and dephosphorization rate. It implies that the mixed injection method using the O_2_ and N_2_ is appropriate for the duplex steelmaking process. 

In the present work, the behavior of a supersonic jet formed by a single Laval nozzle is investigated to explore the feasibility of the O_2_ and N_2_ mixed top-blowing method for the dephosphorization converter. There are five types of Laval nozzle structures investigated, designed with different N_2_ flow rates. Both numerical simulation and experiments are carried out to explore the influence of the N_2_ mixing rate on the supersonic jet behavior. The axial velocity and O_2_ concentration are measured in the experiment to verify the reliability of the simulation model. Meanwhile, the impaction area and the droplet generation rate will be reported to further discuss the impaction ability of the supersonic jet formed by the combination gas.

## 2. Apparatus and Experiment

In this research, based on the one-dimensional isoentropic flow theory, the Laval nozzle structures have been confirmed according to the mixing rate between N_2_ and O_2_. The throat and exit parameters can be calculated by [19]:

(1) $$Q=\frac{60}{\rho_{com}}\sqrt{\frac{\kappa }{R}{\left(\frac{2}{\kappa +1}\right)}^{\frac{\kappa +1}{\kappa -1}}}C_{D}\frac{A_{t}P_{0}}{\sqrt{T_{0}}}$$

(2) $$\frac{A_{t}}{A_{e}}=C_{k}$$

where *Q*, *P_0_*, *T_0_*, and *ρ_com_* arethe flow rate, stagnation pressure, temperature, and density of the combination gas, respectively; *A_t_* and *A_e_* are the throat and exit areas of the Laval nozzle, respectively; *C_D_* is the oxygen utilization coefficient of 0.97; *C_k_* is a constant of 1.6875 based on the design Mach number; *κ* is the ratio of the combination gas heat capacity, which is set equal to 1.4; and *R* is the molar gas constant of 8.314 J·mol^−1^·K^−1^. The Mach number, stagnation pressure, and temperature are 2.00, 0.814 Mpa, and 298 K, respectively. Additionally, the parameters of various Laval nozzle structures are presented in Table 1.

**Figure 1:**
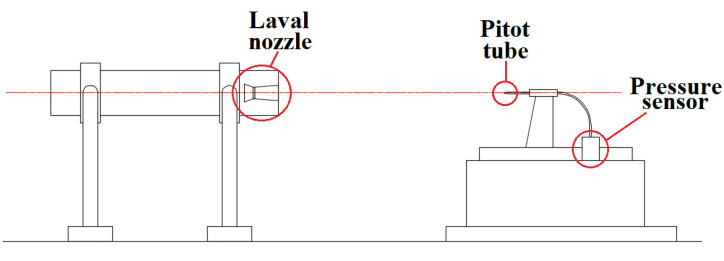
The schematic view of the employed apparatus in the experiment.

Figure 1 presents the schematic view of the apparatus used in the experiment. The oxygen lance with single Laval nozzles was fixed with the metal bracket before the measurement. The pitot tube was positioned at the bedstand and ensured the central point of the nozzle exit, and is collinear for measuring the dynamic and static pressures formed by the combination jet. For achieving the required oxygen concentration, the static pressure hole at the pitot tube was plugged, ensuring that the content of the combination gas was delivered to the flue gas analyzer. Finally, the pitot tube could be replaced by the thermocouple to measure the total temperature of the supersonic jet. In this research work, the experiments were performed at room ambient temperature.

During the measurement process, the Mach number of the combination jet would be in the range of 0.02–2. Therefore, Equations (3) and (4) are adopted to evaluate the axial velocity of the combination gas by measuring the pressure at different positions, referring to the Mach number of the combination gas: [20]

(3) $$V=\sqrt{\frac{2\kappa RT}{\kappa -1}\left[{\left(\frac{P_{s}}{P_{d}}\right)}^{(\kappa -1)/\kappa }-1\right]},\;\mathrm{Ma}\;>\;0.3,$$

(4) $$V=\sqrt{\frac{2(P_{s}-P_{d})}{\rho_{com}}},\;\mathrm{Ma}\;<\;0.3,$$

where, *V*, *T*, *P_d_*, and *P_s_* are the axial velocity, temperature, dynamic, and static pressures of the combination jet, respectively, and the factor *κ* denotes the ratio of the oxygen heat capacity, which is set equal to 1.4.

## 3. Numerical Simulations

### 3.1. Governing Equations

For further analyzing the characteristics of the combination gas flow field, a simulation model calculated by Fluent software has been constructed. The Reynolds averaging formation was adopted in the simulation process to solve the partial differential equations. The Navier–Stokes continuity, momentum, and energy conservation equations are described as follows [21]:

Continuity equation

(5) $$\frac{\partial \rho }{\partial t}+\frac{\partial (\rho \vec{v_{i}})}{\partial x_{i}}=0$$

Momentum conservation equation

(6) $$\frac{\partial (\rho v_{i})}{\partial t}+\frac{\partial (\rho v_{i}v_{j})}{\partial x_{j}}=-\frac{\partial p}{\partial x_{i}}+\frac{\partial \left(\tau_{ij}-\rho \bar{v_{i}^{\prime }v_{j}^{\prime }}\right)}{\partial x_{j}}$$

Energy conservation equation

(7) $$\frac{\partial }{\partial t}(\rho E)+\frac{\partial }{\partial x_{i}}(\rho Ev_{i}+pv_{i}))=-\frac{\partial }{\partial x_{i}}(q_{i}+C_{P}\rho_{i}\bar{v_{i}^{\prime }T^{\prime }})+\frac{\partial (\tau_{ij}v_{j}-\rho_{i}\bar{v_{i}^{\prime }v_{j}^{\prime }}v_{j})}{\partial x_{i}}+S_{h}$$

where *v_i_* and *v_j_* represent the mean velocity components in the *i_th_* and *j_th_* directions, respectively; $v_{i}^{\prime }$ and $v_{j}^{\prime }$ denote the fluctuating velocity components in the *i_th_* and *j_th_* directions, respectively; *S_h_* is the volumetric heat source; and *E* and *τ_ij_* are the total energy and viscous stress, respectively. 

The shear stress transfer (SST) k-w turbulence model is selected in the simulation process because it can accurately represent the diffusivity and dissipation of the supersonic jet. The turbulence kinetic energy (*k*) and its dissipation rate (*w*) in the simulation model are governed by the following relations [22]:

(8) $$\frac{\partial }{\partial t}(\rho k)+\frac{\partial }{\partial x_{i}}(\rho ku_{i})=\frac{\partial }{\partial x_{j}}(\Gamma_{k}\frac{\partial k}{\partial x_{j}})+G_{k}-Y_{k}$$

(9) $$\frac{\partial }{\partial t}(\rho w)+\frac{\partial }{\partial x_{i}}(\rho wu_{i})=\frac{\partial }{\partial x_{j}}(\Gamma_{w}\frac{\partial w}{\partial x_{j}})+G_{w}-Y_{w}$$

where *G_k_* and *G_w_* are the generation of the turbulence kinetic energy due to the mean velocity gradient and the generation of specific dissipation rate, respectively; Γ*_k_* and Γ*_w_* represent the effective diffusivity of *k* and *w*, respectively, and *Y_k_* and *Y_w_* are the dissipation of *k* and *w*, respectively.

### 3.2. Simulation Details

The axial and radial distance for simulation data was measured by the exit diameters of the Laval nozzle to suppress the effect of the Laval nozzle structure on the flow field. In this paper, 1 De was addressed as the exit diameter of the Laval nozzle, and the length of 1 De was changed for each Laval nozzle. For instance, from the results generated by the designed total flow rate of a Laval nozzle of 7000 Nm^3^/h, the length of 1 De was 50.92 mm. 

**Figure 2:**
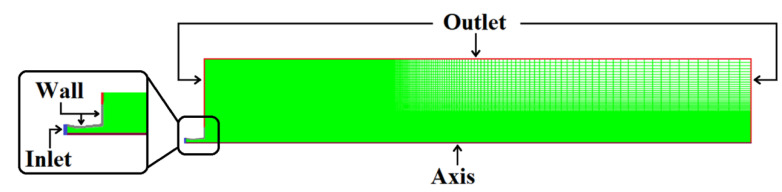
A schematic representation of the mesh model with boundary conditions.

A 2D geometrical model was constructed to investigate the mechanical behavior of the combination gas jet. The Laval nozzle and the combination gas flowing region constituted the computational domain of the simulation model, which was considered to be 65 De downstream in the axial direction from the exit of the Laval nozzle and 10 De in the radial direction. Figure 2 presents the mesh model with boundary conditions schematically. The mesh density near the Laval nozzle region is much higher than other regions due to the higher velocity and the temperature gradient. 

In the initial state, there is no combination gas blowing through the nozzles into the computational domain, such that it is filled with stationary air. An axis boundary condition (modena line) is applied to the computational domain. The mass flow inlet boundary condition (blue line) is chosen at the Laval nozzle inlet for the incoming combination gas. The wall model boundary condition (gray line) is adopted at the oxygen lance wall. A pressure outlet boundary condition (red line) is utilized for other computational domain boundaries. Table 2 shows detailed information on the boundary conditions.

The steady pressure-based solver is adopted to integrate the Navier–Stokes equation with a double-precision option. The pressure-velocity coupling is explicitly solved by the coupled scheme with the second-order spatial discretization for all variables. The solution convergence is determined by two criteria. The first is energy residual <10^−6^ and residuals for other variables <10^−4^. The second criterion is the variation between two successive iterations at the outlet of the computational domain. This is kept within 2.0 K (2 °C) for the average total temperature and 1 m/s for the velocity.

### 3.3. Mesh Independency Test

**Figure 3:**
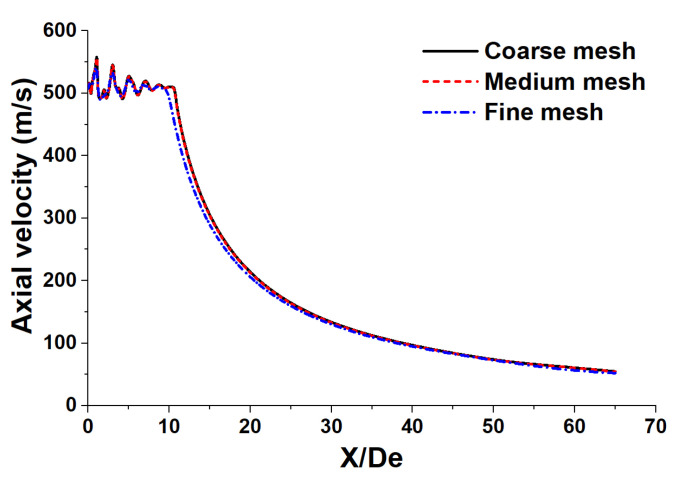
The axial velocity distribution of the supersonic jet at the jet centerline using various mesh levels.

In order to confirm the mesh model sensitivity, numerical simulations are first carried out with three types of mesh level, based on [23], as follows: coarse mesh (73,325 cells), medium mesh (184,580 cells), and fine mesh (299,938 cells). Figure 3 depicts the axial velocity distribution of the supersonic jet at the jet centerline using various mesh levels.

The results show that the average variation of the axial velocity between the coarse mesh and the medium mesh is about 9.7%. For both medium and fine meshes, the axial velocity variation is about 0.3%. This means the simulation results are not sensitive to a mesh in medium and fine mesh cases. However, the required computational time in the case of fine mesh is about 2.7 times longer than that for the medium mesh. Therefore, the medium mesh model has been chosen for analysis and discussion in this research work.

## 4. Results and Discussion

### 4.1. Axial Velocity Distribution

**Figure 4:**
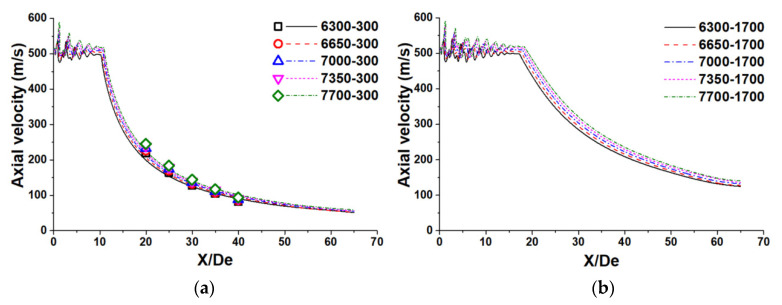
The axial velocity distribution of the supersonic jet at the Laval nozzle centerline: (**a**) room ambient temperature; (**b**) high ambient temperature.

Figure 4 represents the axial velocity distribution of the supersonic oxygen jet at the centerline of the Laval nozzle, with two different ambient temperatures. In the present study, the ambient temperatures of 300 K and 1700 K are considered the room and high ambient temperatures, respectively. Furthermore, the simulation results are depicted by the solid and dotted lines, and the measurement data are presented by various markers (☐, ◯, △, ▽, and ◇). The object property shows the total flow rate and the ambient temperature in Figure 4. For instance, the 7000-300 represents the total flow rate of the combination gas of 7000 Nm^3^/h and the ambition temperature of 300 K. 

Based on the provided results, the average variation of the supersonic jet axial velocity at the Laval nozzle using N_2_ flow rates of 2800, 3150, 3500, 3850, and 4200 Nm^3^/h are 5.6, 5.6, 5.3, 5.1, and 5.0%, respectively, at the room ambient temperature. Furthermore, the average variation of the supersonic jet axial velocity at room ambient temperature for X/De of 20, 25, 30, 35, and 40 are predicted to be 8.5, 6.7, 2.5, 0.6, and 0.7%, respectively. The average variation of the supersonic jet axial velocity commonly reduces with a higher N_2_ flow rate. Meanwhile, this variation first decreases, and then increases with a higher X/De. Hence, the results calculated by the simulation model can represent the axial velocity flow field of the supersonic jet well, which proves the reliability of the proposed simulation model.

At the Laval nozzle exit, a shock train has been formed due to the expansion and oblique shock waves for all types of Laval nozzle structure. As a result, the axial velocity of the potential core will keep fluctuating in a particular range. Such fluctuations reduce as the ratio of X/De grows. The axial velocity of the supersonic jet would then gradually reduce because of the entrainment phenomenon between the combination gas and the ambient flow. For both room and high ambient temperatures, the initial axial velocity of the supersonic jet at the Laval nozzle using N_2_ flow rates of 2800, 3150, 3500, 3850, and 4200 Nm^3^/h are 511.3, 512.3, 513.2, 514.0, and 514.9 m/s, respectively. The viscosity represents the mathematical ratio of the tangential frictional force per unit area to the velocity gradient perpendicular to the liquid flow direction. Thereby, greater resistance is formed for a gas flow with higher viscosity at the same flow velocity. When the ambient temperature and the static pressure are the same, the viscosity of O_2_ is always higher than that of N_2_. With the same pressure variation and divergent section length, the initial axial velocity of the combination gas increases as the N_2_ flow rate grows, and the ambient temperature does not influence the initial axial velocity of the supersonic jet formed by combination gas under the tested conditions.

According to the obtained results, the velocity potential core length of the supersonic jet at room (high) ambient temperature using N_2_ flow rates of 2800, 3150, 3500, 3850, and 4200 Nm^3^/h are 10.3 (17.6) De, 10.6 (18.2) De, 10.8 (18.7) De, 11.0 (19.2) De, and 11. (19.7) De, respectively. The length of the potential core at a high ambient temperature based on N_2_ flow rates of 2800, 3150, 3500, 3850, and 4200 Nm^3^/h are 1.71, 1.72, 1.73, 1.75, and 1.76 De times larger, respectively, than at room ambient temperature. As mentioned in Table 1, the exit diameter of the Laval nozzle reduces as the N_2_ flow rate increases. This means the initial diameter of the surface area between the combination gas and the ambient flow in the case of the N_2_ flow rate with 4200 Nm^3^/h is smaller than the other cases. As a result, the entrainment phenomenon would be suppressed due to a smaller exit diameter, causing resistance to the combination gas for a higher N_2_ flow rate at the nozzle exit. As a result, a Laval nozzle designed with a greater N_2_ flow rate can prolong the velocity potential core length such that a higher ambient temperature is able to improve this trend further.

**Figure 5:**
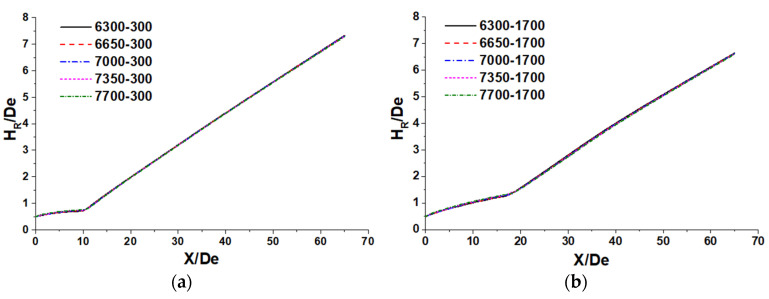
Half-jet radius distribution of supersonic jet with various N_2_ flow rates: (**a**) room ambient temperature; (**b**) high ambient temperature.

The half-jet radius (H_R_) is denoted as the radial distance from the supersonic jet centerline, where the velocity of the jet becomes half of the axial velocity. Figure 5 shows the half-jet radius of the supersonic jet with various N_2_ flow rates at room and high ambition temperatures. At both room and high ambient temperatures, the H_R_ increases slowly until it reaches the axial velocity potential core and then grows at a higher rate. By increasing the N_2_ flow rate, this flow pattern is more-or-less preserved. The segment that is the range from 0D_e_ to the end of the potential core is defined as the A segment, and the line segment that is the range from the end of the potential core to 65 De is defined as the B segment. Based on the results, the slopes of the A and B segments are given in Table 3.

The obtained results indicate that both the A and the B segment slopes increase for high ambient temperatures and reduce as the N_2_ flow rate increases. After the combination gas reaches the potential core, the velocity of the combination gas quickly decreases, suppressing the diffusion rate of the combination gas due to a smaller velocity gradient. Therefore, the ambient temperature has a higher impact on the A segment slope than on the B segment slope. As reported in [24], the theoretical spreading rate for a free turbulent jet is 0.1, whereas the average slope of the B segment at high ambient temperatures is 0.1241, and this simulation data at room ambient temperature is 0.1190. This means the supersonic combination gas needs a longer developing length to be a free turbulent jet at a higher ambient temperature.

### 4.2. Oxygen Distribution and Utilization Rate

**Figure 6:**
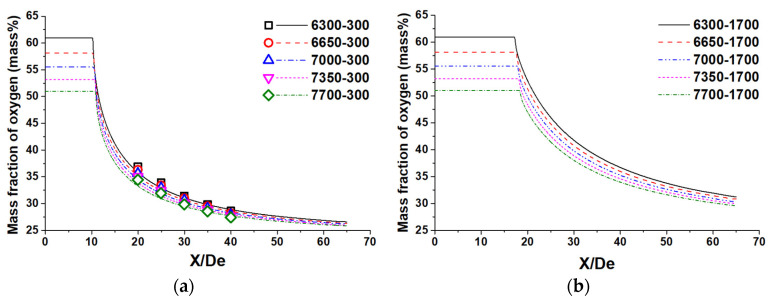
The oxygen distribution of the supersonic jet at the Laval nozzle centerline: (**a**) room ambient temperature; (**b**) high ambient temperature.

Figure 6 depicts the oxygen distribution of the supersonic oxygen jet at the centerline of the Laval nozzle at room and high ambient temperatures. The oxygen mass fraction is kept unchanged within the axial velocity potential core, and it then gradually reduces. As shown in Figure 6a, there exists a reasonably good agreement between the theoretically predicted results and these measured data. For analyzing the oxygen diffusion rate, the oxygen diffusion length of the supersonic jet is defined as the distance between the end of the axial velocity potential core and the point of the reduction rate of the oxygen mass fraction being 1.0.

Based on the obtained results, the average variation of the O_2_ content at the Laval nozzle using N_2_ flow rates of 2800, 3150, 3500, 3850, and 4200 Nm^3^/h are 1.63, 1.78, 1.82, 1.85, and 1.86%, respectively, at room ambient temperature. Further, the average variation of the O_2_ content at the X/De of 20, 25, 30, 35, and 40 are 3.3, 3.2, 1.1, 0.2, and 0.1%, respectively, at room ambient temperature. The average variation of the O_2_ content increases as the N_2_ flow rate becomes higher. Meanwhile, this variation keeps reducing as the ratio X/De lessens. Therefore, the simulation results are in good agreement with the measurement data for O_2_ content at room ambient temperature, which further proves the accuracy of the simulation model proposed in this paper.

The oxygen diffusion length at the room ambient temperature using N_2_ flow rates of 2800, 3150, 3500, 3850, and 4200 Nm^3^/h are 7.4 De, 6.8 De, 6.2 De, 5.8 De, and 5.3 De, respectively. At a high ambient temperature, the measured factors mentioned above for these N_2_ flow rates are 8.2 De, 6.8 De, 5.8 De, 4.7 De, and 3.6 De, respectively. As mentioned, by increasing the N_2_ flow rate, the viscosity of the combination gas decreases. Although combination gas with a smaller viscosity can be further accelerated at the same pressure gradient, its diffusion rate would be higher than that of a combination gas with a greater viscosity when the combination gas passes through the still ambient gas. As a result, the oxygen diffusion length reduces as the N_2_ flow rate magnifies. The oxygen diffusion length at a high ambient temperature using N_2_ flow rates of 2800, 3150, 3500, 3850, and 4200 Nm^3^/h are, respectively, 1.11, 1.00, 0.94, 0.81, and 0.68 De times larger than that at the room ambient temperature. This means that, at high ambient temperatures, the oxygen diffusion length will first be prolonged and then reduced using a greater N_2_ flow rate.

**Figure 7:**
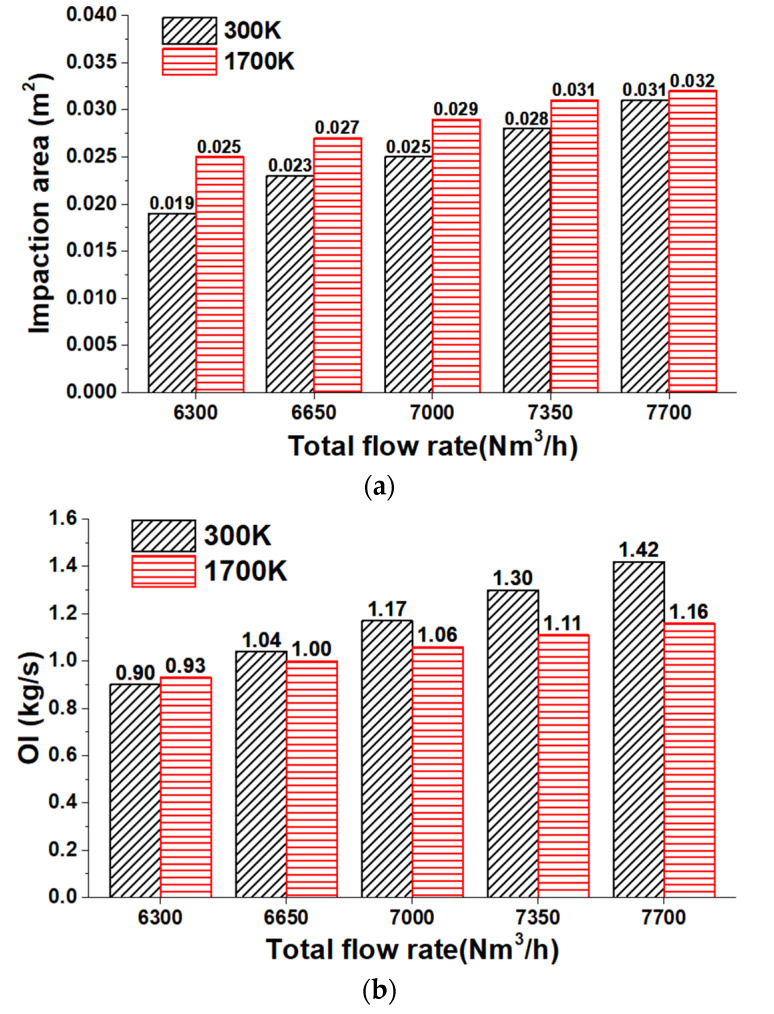
The impact area and the OI distributions at a lance height of 1550 mm: (**a**) impact area; (**b**) OI.

As discussed in Section 4.1, a greater N_2_ flow rate improves the axial velocity of the potential core and increases the diffusion rate of the combination gas. To investigate the oxygen utilization rate in detail, the oxygen flow rate at the impaction area, abbreviated as OI, is calculated. The impact area is defined as the region in which the oxygen jet can penetrate the slag layer and directly contact the molten steel at a certain lance height. The lance height and thickness of the slag layer are 1550 and 250 mm, respectively. As reported in [25], the following relation would be helpful when the oxygen jet has just penetrated the slag layer:

(10) $$P_{g}-P_{s}=\frac{\rho_{g}v_{g}^{2}}{2}-\rho_{s}gh_{s}=0$$

where *P_g_* and *v_g_* denote the dynamic pressure and velocity of the combination gas, respectively, *ρ_g_* and *ρ_s_* present the densities of the combination gas and slag layer, respectively, and *P_s_* is the dynamic pressure of the combination gas that penetrates the slag layer without having any impact on the molten steel. Hence, the dynamic pressure of the combination gas is considered to be 7358 Pa for evaluation of the impact area. Figure 7 shows the impact area and the OI distribution at the lance height of 1550 mm.

The results show that both impact area and OI increase by increasing the N_2_ flow rate at both room and high ambient temperatures. The impact area at a high ambient temperature by considering the total flow rate of 6300, 6650, 7000, 7350, and 7700 Nm^3^/h are, respectively, 1.14, 1.10, 1.07, 1.04, and 1.02 times larger than at room ambient temperature. Hence, the impact area grows with the ambient temperature, but this increasing rate of impact area reduces by increasing the total flow rate, as demonstrated in Figure 7a,b, which shows that OI at a high ambient temperature based on the total flow rate of 6300, 6650, 7000, 7350, and 7700 Nm^3^/h would be, respectively, 1.04, 0.96, 0.90, 0.86, and 0.82 times larger than that at the room ambient temperature. The recently obtained results confirm that, by increasing the total flow rate, the OI first increases and then gradually reduces. This means a higher ambient temperature suppresses the OI, except at the total flow rate of 6300 Nm^3^/h. Therefore, a higher N_2_ flow rate can expand the oxygen utilization rate at room and high ambient temperatures; nevertheless, a higher ambient temperature suppresses this.

### 4.3. Droplet Generation

**Figure 8:**
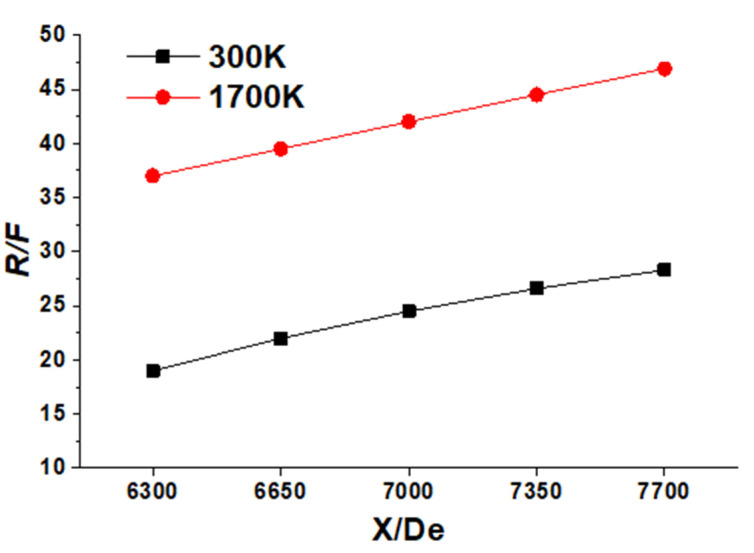
The *R/F* distribution with a lance height of 1550 mm at the Laval nozzle centerline.

To study the effect of the combination gas flow rate on the droplet generation rate, the blowing number (*N_B_*) of the combination gas at various operation conditions [26] is calculated from the following relation:

(11) $$N_{B}=\frac{\rho_{g}\eta^{2}v_{a}^{2}}{2\sqrt{\sigma g\rho_{s}}}$$

where *ρ_g_* and *ρ_s_* are the density of the combination gas and the molten steel, resectively, and *v_a_* represents the axial velocity of the combination gas. The parameters *σ* and *η* are the surface tension of the molten steel of 1.7 and the constant of value 0.44721, respectively. Based on the definition of *N_B_*, the droplet generation rate per unit volume of the combination gas can be calculated as:

(12) $$\frac{R}{F}=\frac{{\left(N_{B}\right)}^{3.2}}{{\left[2.6\times 10^{6}+2.0\times 10^{-4}{\left(N_{B}\right)}^{12}\right]}^{0.2}}$$

where *R* and *F* are the droplet generation and the volumetric flow of the combination gas, respectively. Dogan et al. [11] reported that the kinetic energy has the biggest impact on droplet generation at the molten bath’s surface, compared to the changes in the molten steel properties. Hence, the variations of the molten surface tension formed by the temperature and composition have been ignored in this research. Figure 8 shows the *R/F* distribution with a lance height of 1550 mm at the Laval nozzle centerline. 

The results show that the ratio of *R/F* improves with the total flow rate and ambient temperature. The *R/F* at a high ambient temperature for the total flow rate of 6300, 6650, 7000, 7350, and 7700 Nm^3^/h are, respectively, 1.95, 1.80, 1.71, 1.67, and 1.66 times larger than that at the room ambient temperature. Therefore, the *R/F* increases with the ambient temperature, but a greater total flow rate will suppress this increasing rate of the *R/F*. At a (high) room ambient temperature, the slopes of the *R/F* graphs as per the 350 total flow rate in the total flow rate range of 6300–7700 mm are 0.158 (0.068), 0.114 (0.063), 0.086 (0.060), and 0.064 (0.054) h/(Nm^3^), respectively. It seems that the increasing rate of the R/F reduces as the total flow rate grows at both room and high ambient temperature; however, this issue is more evident at high ambient temperatures.

## 5. Conclusions

The mechanical behavior of the single supersonic jet formed by various combination gas flow rates was explored by a series of experiments and numerical simulations. The accuracy of the simulation model was then proven by comparing the measured data of the axial velocity and the oxygen content at the Laval nozzle centerline with those of the proposed numerical model. The main results obtained are summarized as follows:

(1) The potential core length of the axial velocity is prolonged as the flow rate of combination gas and ambient temperature grows. For a higher N_2_ flow rate, the viscosity of the combination gas is suppressed, resulting in a higher initial axial velocity at the exit of the Laval nozzle.
(2) The ambient temperature has more influence on the segment slope of the A segment slope than that of the B segment for the half-jet radius of the supersonic jet because the velocity gradient variation at the A segment is greater than that at the B segment.
(3) Compared with room ambient temperature, the oxygen diffusion length is prolonged at the initial stage and then reduces as the N_2_ flow rate grows at high ambient temperatures.
(4) Although the impact area increases with the ambient temperature, its rate would reduce as the total flow rate magnifies. Meanwhile, a growth of the N_2_ flow rate leads to the improvement of the oxygen utilization rate at different ambient temperatures; however, this issue is diminished at higher ambient temperatures.
(5) The increasing rate of the R/F becomes lower for higher levels of the total flow rate, and such an increasing rate is further suppressed by a higher ambient temperature.

The present investigation reveals the initial efforts to understand the combination gas top-blowing method in the duplex steelmaking process. This paper shows that the axial velocity, impact area, and oxygen utilization rate can be improved by exploiting higher flow rates of the combination gas at the same oxygen flow rate. This fact also proves the feasibility of the combination gas top-blowing method. Therefore, more experiments and numerical simulations on the flow field of the molten bath and supersonic jets formed by multi-nozzles should be performed to see whether this technology could be adopted for the basic oxygen furnace (BOF) steelmaking process. Subsequently, the gas supply method, slagging control process, and dust removing system should be redesigned to use the combination gas top-blowing method. Finally, compared with the traditional oxygen supply method, the operating cost of the combination gas top-blowing method should be calculated to find a suitable operational model.

## Figures and Tables

**Table 1:** The parameters of various Laval nozzle structures.

Design Total Flow Rate (Nm^3^/h)	Design N_2_ Flow Rate (Nm^3^/h)	Design O_2_ Flow Rate (Nm^3^/h)	Throat Diameter (mm)	Exit Diameter (mm)	Divergent Section Length (mm)
6300	2800	3500	39.36	51.12	80.00
6650	3150	3500	39.28	51.02	80.00
7000	3500	3500	39.20	50.92	80.00
7350	3850	3500	39.14	50.84	80.00
7700	4200	3500	39.08	50.78	80.00

**Table 2:** The detailed information of the boundary conditions.

Name of Boundary	Type of Boundary Conditions	Values
Main oxygen inlet	Mass flow rate	2.2778 kg/s, 2.3889 kg/s, 2.5000 kg/s, 2.6111 kg/s and 2.7222 kg/s
	O_2_ mass fractions	61.0%, 58.1%, 55.6%, 53.2% and 51.0%
	N_2_ mass fractions	39.0%, 41.9%, 44.4%, 46.8% and 49.0%
	Oxygen temperature	298 K
Outlet	Static pressure	104,000 Pa
	Mass fractions	O_2_ = 23%, N_2_ = 77%
	Ambient temperature	300 K, 1700 K

**Table 3:** The slopes of the A and B segments.

Total Flow Rate (Nm^3^/h)	Room Ambient Temperature		High Ambient Temperature	
	A	B	A	B
6300	0.0299	0.1193	0.0513	0.1246
6650	0.0290	0.1191	0.0497	0.1244
7000	0.0282	0.1190	0.0485	0.1242
7350	0.0271	0.1188	0.0475	0.1239
7700	0.0264	0.1186	0.0465	0.1236

## Data Availability

Not applicable.
